# Evaluating dose response from flexible dose clinical trials

**DOI:** 10.1186/1471-244X-8-3

**Published:** 2008-01-07

**Authors:** Ilya Lipkovich, David H Adams, Craig Mallinckrodt, Doug Faries, David Baron, John P Houston

**Affiliations:** 1 Lilly Research Laboratories, Eli Lilly and Company, Indianapolis Indiana, USA; 2 Department of Psychiatry and Behavioral Sciences, Temple University School of Medicine, Philadelphia Pennsylvania, USA

## Abstract

**Background:**

The true dose effect in flexible-dose clinical trials may be obscured and even reversed because dose and outcome are related.

**Methods:**

To evaluate dose effect in response on primary efficacy scales from 2 randomized, double-blind, flexible-dose trials of patients with bipolar mania who received olanzapine (N = 234, 5–20 mg/day), or patients with schizophrenia who received olanzapine (N = 172, 10–20 mg/day), we used marginal structural models, inverse probability of treatment weighting (MSM, IPTW) methodology. Dose profiles for mean changes from baseline were evaluated using weighted MSM with a repeated measures model. To adjust for selection bias due to non-random dose assignment and dropouts, patient-specific time-dependent weights were determined as products of (i) stable weights based on inverse probability of receiving the sequence of dose assignments that was actually received by a patient up to given time multiplied by (ii) stable weights based on inverse probability of patient remaining on treatment by that time. Results were compared with those by unweighted analyses.

**Results:**

While the observed difference in efficacy scores for dose groups for the unweighted analysis strongly favored lower doses, the weighted analyses showed no strong dose effects and, in some cases, reversed the apparent "negative dose effect."

**Conclusion:**

While naïve comparison of groups by last or modal dose in a flexible-dose trial may result in severely biased efficacy analyses, the MSM with IPTW estimators approach may be a valuable method of removing these biases and evaluating potential dose effect, which may prove useful for planning confirmatory trials.

## Background

Knowledge of the relationship between drug dose and clinical response contributes to the safe and effective use of medications. Clinical drug trials using double-blind, parallel, randomized assignment to fixed-dose groups are considered the gold standard for evaluating dose response for clinical outcomes both in exploratory and confirmatory phases of drug development. In fixed dose trials, interpretation of statistical inference can be done in terms of causal relationship between treatment and an outcome, based on the principle of randomization.

In examining dose response for long-term outcomes, fixed dose trials have several limitations including maintaining a patient on a possibly suboptimal dose or a dose with intolerable side-effects, poor comparability to actual clinical practice, and restrictive inclusion/exclusion criteria. This is exacerbated by the wide variation between individual patients in pharmacokinetic and pharmacodynamic profiles found with many medications. Not surprisingly, fixed-dose trials, especially in neuroscience, suffer from high discontinuation rates. High discontinuation rates may result in biased or inefficient inference and subsequent conclusions, especially if different dose groups exhibit different discontinuation patterns. Likelihood-based approaches allow adjustment for dropouts explicitly (multiple imputation – MI) or implicitly (mixed-effects model, repeated measures – MMRM) and typically result in less biased estimates of treatment effects than the popular last observation carried forward (LOCF) approach [1].

Flexible dose trials are better at mimicking actual clinical practice and better reflect risk/benefit considerations since dose may be changed in accordance with individual patient response. It would be of great scientific and clinical value if dose response relationships could be evaluated from flexible dose trials. When employing a flexible regimen, dose is typically assigned based on previously observed outcomes (efficacy/tolerability) and direct comparison of dose groups at any time or overall is subject to selection bias (e.g. the patients who received the highest dose at the last scheduled visit may show less improvement than patients who end up on the lowest dose, since the former are typically assigned to the less responsive patients). This is similar to the selection bias in comparison of treatment (dose) groups using only data from patients who remained on treatment by specific endpoint. In a sense, switching treatment, adjusting dose, and discontinuing a patient involve decisions that may cause selection bias.

Robins and colleagues [2,3] and Hernán and colleagues [4-6] proposed and implemented, in the context of observational clinical trials, a methodology of adjusting for selection bias caused by non-random treatment switching very similar to inverse-probability-of-censoring weighting used to adjust for bias caused by missing values due to dropout when estimating treatment effect from longitudinal data [7]. In their approach [2-6], based on inverse-probability-of-treatment weighting (IPTW), treatment comparisons are conducted on the pseudo-population, re-weighted inversely to the estimated probability of patients receiving the treatment sequence they actually received by any given time point. Because this approach leads to the evaluation of *marginal *(unconditional on past outcome) means of *potential *outcome for any given treatment sequence, thus revealing the causal mechanism (or the "structure") behind the observed data, it was termed by the authors "*marginal structural *models" (MSMs).

In the present study, we used the MSM approach to evaluate dose response relationship in flexible dose trials, considering dose adjustment a special case of treatment switching. The goal was to adjust for selection bias in dose effect caused by non-random mechanism of dose assignment by (1) assessing this mechanism using a statistical model for probability of dose assignment, and (2) relating outcome to a recent and past dose using standard statistical procedures adjusted for selection bias with weights, based on inverse probability of the dose sequence that was actually observed (estimated at Step 1). As a result, it was possible to evaluate the *potential *efficacy of higher dose versus lower dose by evaluating difference in potential (or *counterfactual*, [8]) outcomes predicted by the MSM for a hypothetical patient who would have been assigned to a higher versus a lower dose throughout the entire trial. Using this approach, we analyzed dose relationships from 2 flexible-dose trials of antipsychotics in the treatment of schizophrenia and bipolar disorder.

## Methods

### Clinical trial designs

To investigate dose relationships in flexible dose trials, we analyzed data from olanzapine-treated patients from 2 randomized, double-blind clinical trials with flexible dosing. The baseline characteristics of patients included in these trials are summarized in Table 1. Study A [9] was a 12-week study of acutely-ill, bipolar I patients with an index manic episode (N = 452) who received olanzapine (5, 10, 15, 20 mg/day, n = 234) or haloperidol. Study B [10] was a 28-week study in acutely ill patients with schizophrenia, schizophreniform disorder, or schizoaffective disorder (N = 339) who received olanzapine (10, 15, 20 mg/day, n = 172) or risperidone. In both studies, investigators made dose increases or decreases as clinically indicated; while they were blinded with respect to the actual treatment, they were aware of the dose level as expressed by the number of capsules prescribed. Placebo capsules were used so that an equivalent number of capsules were given regardless of the assigned drug. The dose could be adjusted upward one increment at a time, or could be reduced by one or more decrements at a time. Concomitant use of anticholinergics or benzodiazepines was allowed for treatment-emergent symptoms of extrapyramidal symptoms (EPS) or agitation. For each of the clinical trials, the protocol was approved by ethical review boards responsible for study sites and all patients gave written, informed consent prior to entering the study.

**Table 1:** Study characteristics

	Study A N* = 234	Study B N* = 172
Disease state	Bipolar I mania	Schizophrenia
Duration of study	12 weeks	28 weeks
Visit intervals	2 weeks	2–4 weeks
Dosages, mg/day	Olanzapine (5, 10, 15, 20)	Olanzapine (10, 15, 20)
Starting dose, mg/day	Olanzapine (15)	Olanzapine (15)
Gender, % female	60.2	35.1
Age in years, mean (SD)	39.9 (13.2)	36.2 (10.7)
Symptom severity, mean (SD)	YMRS total, 30.7 (7.5)	PANSS total, 96 (16.6)

*Olanzapine-treatment arm only

### Variables and measures

Since, in both studies, dose of medication could change at or sometimes between evaluation visits, for this analysis, dose was summarized with a single value per visit interval calculated as the most frequent (modal) dose received by a patient after an evaluating visit up to and including the next evaluation visit (interval modal dose). To facilitate comparisons for high versus low dose and avoid groups with a small number of subjects, these values were further grouped as follows: for the bipolar I study, 5–10 mg, 11–15 mg, and 16–20 mg olanzapine dose groups, and for the schizophrenia study, 10–15 mg and 16–20 mg olanzapine dose groups.

Therapeutic efficacy in the bipolar I study was evaluated using the Young Mania Rating Scale (YMRS) [11]. In the schizophrenia study, efficacy was measured with the Positive and Negative Syndrome Scale (PANSS) [12].

### Statistical methodology

#### Unweighted analyses

Two unweighted analyses were performed: (i) "naïve analysis" using separate analysis of covariance (ANCOVA) models (at every visit interval) for reduction in symptoms from baseline as the dependent variable with terms for modal dose during previous visit interval and baseline efficacy score, and (ii) likelihood-based MMRM fitted to reduction in symptoms at every evaluation visit with terms for time-varying dose (during the previous interval), the current visit interval, visit by dose interaction, baseline score, and baseline score by visit interaction. The dependency in repeated observations was modeled using an unstructured covariance fitted to within-patient errors.

#### Weighted analysis (marginal structural models)

To evaluate differences between different dose levels, we implemented a 2-step IPTW MSM scheme.

##### (1) Construction of weights

We applied an ordinal logistic regression model (proc GENMOD in SAS 8.02) to data pooled from all the patients records (i.e. considering each patient-visit as an "independent" observation) within each trial to estimate probabilities of receiving a dose that was actually received by each patient during each visit interval, given the recent history of dosage and past treatment outcome (see Table 2). The dependent variable was the dose received during the current visit interval, and the predictors were dose during the previous visit, changes in disease severity (efficacy) score from baseline to the end of previous visit interval, maximal severity of any adverse event (AE) that occurred during the previous visit interval, and efficacy score at baseline. Then, probability of dose sequence received up to a given visit interval was computed as a product of conditional probabilities for all the previously assigned dosages starting from the first post-baseline visit interval. Subject-specific weights at every time point can be computed simply as inverse of this "cumulative" probability. However, since such weights typically exhibit high variability and may lead to an unduly large impact on the estimates by only a few observations, Robins *et al.*[3] proposed and Hernán, *et al.*[4] implemented a modified version of weights called "stabilized weights." In our analyses, we closely followed their methodology and therefore interested readers should refer to these publications for details. Stabilized weights were constructed as the ratio of two estimates for probability *of the dose sequence received up to a given time: *the estimate in the denominator was based on the model with previous dose, baseline disease severity scores, and potential time-dependent confounders: previous efficacy scores and AE severity indicator; the estimate in the numerator was based on a similar model, except all potential time-dependent confounders were excluded.

**Table 2:** Modeling probability of having a higher dose, given previous treatment, outcome history, and baseline score

**Predictors of dose level during visit interval (adjusted for previous dose level)**	**Study A**		**Study B**	
	
	odds ratio* (95% CI)	**p-value**	odds ratio* (95% CI)	**p-value**
YMRS** (Study A) or PANSS** (Study B) reduction from baseline to the end of previous visit interval	0.88 (0.86, 090)	< .0001	0.97 (0.96–0.99)	<.0001
Adverse event (AE) indicator (max severity score during previous visit interval)	0.87 (0.74–1.01)	.075	1.02 (.78–1.33)	ns
Baseline YMRS** (Study A) or PANSS** (Study B) total score	1.14 (1.10, 1.18)	< .0001	1.04 (1.02–1.05)	<.0001

*Estimated by applying the ordinal logistic regression model with the dependent variable as dose received during the current visit interval and the listed predictors. Previous dose was fit into the model as a categorical variable, with associated coefficients capturing effects of specific dose level versus "reference" dose (results were highly significant as to be expected and are not shown here for the sake of the space).

** YMRS total scores range from 0–60. PANSS total scores range from 30 to 210.

In addition to stabilized weights accounting for dose switching as described above, weights accounting for selection bias due to dropouts were constructed in a similar fashion, based on the inverse probability of a patient remaining in the study (remaining uncensored) by a given time. The latter was estimated by multiplying the conditional probabilities of patient remaining uncensored by every time point up to a given time. The conditional probabilities were estimated by pooled (repeated) logistic regressions with an indicator for patient presence/absence in the study at every time interval following evaluation visit (up to discontinuation or study completion) as a dependent variable and current dose, severity of AE, and efficacy scores during current and previous visit intervals as independent variables. The final weights accounting for selection biases, both due to dose switching and dropping-out, were computed as the product of these two weights.

##### (2) Fitting weighted MSM models

We used estimated weights in causal inference of dose effect on subsequent efficacy outcome conducted with a pooled (repeated measures) linear MSM model. The essence of MSM is that by using IPTW a simple association model that connects the *observed *outcome at any time point with *observed *dose history at previous time points can be turned into a causal model that connects potential outcome at that time point with a pre-specified dose history (see Hernán *et al.*[6], Section 5). More specifically, our model for potential outcome expected under dose history to time *t*, H_t-1_(D), was:



where the dependent variable ΔY_t _= Y_0_-Y_t_, was the amount of reduction in severity score from baseline to the end of visit interval *t*, (starting from the 3rd post-baseline visit). The effects of time, baseline disease severity, and dose on the outcome were captured as follows:

• "time" effect was modeled via visit-specific intercept α(t);

• effect of baseline score (*Y*_0_) modeled via time-fixed coefficient γ_5_; a more general model was actually evaluated by incorporating visit-specific coefficients γ_5_(t) allowing the effect of baseline score to vary over time;

• effect of current dose and recent dose history on the outcome measured at end of each visit interval was modeled by estimating effects for the current dose (*d*_t-1_), two previous doses (*d*_t-2_, *d*_t-3_) and average dose during all visits earlier than *t*-3, (${\bar{d}}_{t-4}$), rounded to the category corresponding to the closest dose interval. For all dose variables, we used "grouped dose" as explained in the Methods section. The model does not constrain effects of specific dose levels to be linear or of any parametric form, but (like the effect of visit interval) is left unspecified assuming a unique possible effect of every dose level, captured by separate terms as shown in equation (1). For example, let us assume that doses are grouped into 3 levels, *low*, *medium*, and *high*, designated as indices {1,2,3}. Then the effects of current dose are captured by model terms γ_1_(1), γ_1_(2), and γ_1_(3). As always for identifiability one of these effects should be set to 0 (as done internally in PROC GENMOD). For example, if the lower dose effect γ_1_(1) = 0, then the terms, γ_1_(2) and γ_1_(3) capture the effects of medium and high doses respectively, relative to the lower dose (as the reference category).

• additional *interaction terms *were included in model (1) to capture possible difference in dose effect across time, whenever found significant. Also as was already explained, interaction between baseline value and visit, allowing for time varying effect of baseline severity, was included in the model as it was significant in most of the cases.

The parameters of the model were estimated using weighted generalized estimating equations with normal errors, identity link, independent working correlation structure, and robust sandwich estimator of standard errors (Proc GENMOD in SAS 8.02).

**Figure 1:**
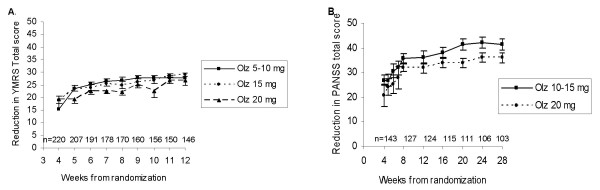
**Marginal structural model with IPTW**. **A**. Cumulative potential dose effect profiles by dose groups for Study A (each dose profile is estimated from MSM model for a hypothetical patient of average baseline severity who would have continuously received the same dose throughout the study); estimated from marginal structural model with IPTW for reduction in the Young Mania Rating Scale (YMRS) total score in patients with bipolar disorder. Olanzapine (Olz): For 20 vs. 5–10 mg, overall dose effect NS; significant negative dose effect (p < .05) at Weeks 4, 7, 8, 10. **B**. Cumulative potential dose effect profiles by dose groups for Study B (each dose profile is estimated from MSM model for a hypothetical patient of average baseline severity who would have continuously received the same dose throughout the study); estimated by marginal structural model with IPTW for reduction in the Positive and Negative Syndrome Scale (PANSS) total score in patients with schizophrenia. Olanzapine (Olz): no significant overall dose effect; significant negative dose effect (p < .05) at Weeks 20, 24.

Assuming that (*i*) all relevant time-dependent confounders were correctly accounted for in estimating probabilities for dose assignments and for patient discontinuation, and that (*ii*) the MSM model was correctly specified, the evaluation of dose-response relationship from MSM allows for causal interpretation [3]. Using expression (1) we can estimate the potential outcomes over time (dose profiles) for any given pre-specified dose sequence and baseline severity (Y_0_). For example, if we are interested in estimating causal effect (by time *t*) of continuously receiving high versus low dose by an average patient all we need to do is to (*i*) evaluate the expected outcomes under high dose by plugging dose = 3 (the code for *high *level) in all terms involving dose in equation (1), including the interaction terms if present; (*ii*) then do the same for dose = 1 (*low)*, and subtract one outcome from the other. Assuming (for simplicity) that there are no interaction terms, the causal dose effect would be constant over time and simply equal to the sum of estimated coefficients γ_1_(3) + γ_2_(3) + γ_3_(3) + γ_4_(3), as all other terms not involving dose will cancel out. If any *dose by time *interaction terms are present, dose effect would change over time. The significance of any such causal effect can then be easily evaluated by testing a hypothesis that the sum of the parameters as shown above is different from zero. The expected potential outcomes for a hypothetical patient at specific level of baseline severity who would have been continuously assigned the same dose (*d*) throughout the study can be estimated (and associated 95% confidence intervals and p-values can be obtained) from the output of proc GENMOD by performing inference on linear predictors at specific values of predictor variables (e.g. by creating a data set with such hypothetical records of subjects whose baseline covariates are fixed at their mean levels and the current and all previous doses fixed at level *d *and passing it to GENMOD for evaluation). To account for the correlation in repeated measures and the fact that the subject weights depend on previous data points we used robust conservative estimates of standard errors. Examples of such estimated potential outcomes are given in Figure 1.

As a part of sensitivity analysis we fitted various other baseline covariates in both equation (1), and models for evaluating weights, as such covariates could potentially be considered by clinicians in dose assignment, and also could have an effect on outcome. However, only baseline severity proved a significant covariate.

An MSM very similar to the one described in equation (1) was tested on simulated data mimicking flexible dose trials for various realistic scenarios (including some probabilities close to 0 to model abrupt changes of dose between 2 visit intervals), involving both positive dose effect and no dose effect in presence of time-dependent confounding. The results indicated a good ability of the model to recover the true dose effect [13]. Also a conceptually similar MSM with lagged predictors was considered by Diggle *et al.*[14].

## Results

### Study A

**Figure 2:**
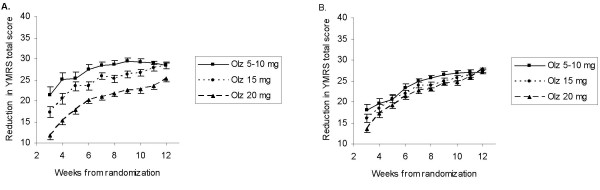
**Unweighted analysis**. **A**. Least-squares means in groups by modal dose received during the most recent visit interval; estimated from unweighted analysis of covariance for reduction in the Young Mania Rating Scale (YMRS) total score in patients with bipolar disorder. Olanzapine (Olz): negative dose effect (20 vs.5–10 mg) at Weeks 3–12, p < .01. **B**. Least-squares means in groups by modal dose received during the most recent visit interval; estimated from unweighted mixed-effects model, repeated measures (MMRM) analysis of reduction in the Young Mania Rating Scale (YMRS) total score in patients with bipolar disorder. Olanzapine (Olz): overall dose effect (20 vs. 5–10 mg) for Weeks 3–12 is negative, p < .001; individual dose contrasts significant (p < .05) at Weeks 3,7,8,10.

Naïve, observed case ANCOVA showed an apparent negative dose effect, that is, less response at higher doses for olanzapine (p < .01) in patients with bipolar disorder (Figure 2A). The magnitude of the negative dose effect observed with MMRM analysis with dose as a time-varying covariate was less than in the observed case model, but was still significant for olanzapine (negative dose effect, p < .001) (Figure 2B). Using MSM IPTW methodology, in Figure 1 we presented dose profiles for every sequence of doses fixed at one of the levels for a patient with "average" disease severity at baseline. No significant overall (i.e. effect estimated over entire study) dose effect on response was estimated for olanzapine. However, there was a significant negative effect (20 vs. 5–10 mg) remaining at Weeks 4, 7, 8, 10 which attenuated by the end of the study (Figure 1A).

### Study B

In the study of patients with schizophrenia, ANCOVA showed an apparent negative dose effect for olanzapine (p < .05), with less response at higher doses, similar to the observed case analysis in Study A. MMRM analysis with dose as a time-varying covariate did not reveal an overall significant dose effect. Using IPTW methodology, there was no significant overall dose effect for olanzapine, but a significant negative dose effect at Weeks 20 and 24 remained (Figure 1B).

In order to compare our findings from MSM analyses of flexible dose trials with fixed dose trials of the same antipsychotic medications (the gold standard), a summary of fixed dose studies of olanzapine in schizophrenia and bipolar disorder is presented in Table 3.

**Table 3:** Dose response findings from fixed-dose randomized trials of olanzapine reported in the literature

**Disease state**	**Study design**	**Duration**	**Dose**	N*	**Dose response for efficacy outcome**
					**Yes**

Chronic schizophrenia [16]	Double-blind, randomized	6 weeks	5, 10, or 15 mg/d	198	Positive linear trend
Chronic schizophrenia [17]	Double-blind, randomized	6 weeks	5, 10, or 15 mg/d	350	Positive linear trend

					**No**

Chronic schizophrenia or schizoaffective disorder [18]	Double-blind, randomized,	24 weeks	10, 15, or 20 mg/d	202	No significant trend
Schizophrenia or schizoaffective disorder [19]	Double-blind, randomized	8 weeks	10, 20, or 40 mg/d	599	No significant dose response

## Discussion

IPTW may provide a methodology to estimate a dose response relationship from flexible-dose clinical trials. IPTW analysis of 2 flexible-dose trials of antipsychotics suggested that within the dose ranges examined, there was no strong negative or overall positive dose effect of the examined antipsychotic medications.

The International Conference on Harmonization of Technical Requirements for Registration of Pharmaceuticals for Human Use (ICH) guidelines for drug development state that, in addition to seeking dose-response information from studies specifically designed to provide it, the entire database should be examined intensively for possible dose-response effects [15]. MSM with IPTW estimators [3,4] may be a method by which additional dose response information can be obtained. By this approach, the probability of having been assigned the dose sequence (plan) that was actually received is estimated for every patient at each time point based on previously observed outcomes and dosage history. Patients who had larger estimated probabilities of being treated with the dose plan they actually received are considered over-represented (compared to a population from a hypothetical trial where patients would have been sequentially re-assigned at every evaluation visit to a dose group by chance) and are penalized by assigning smaller weights, whereas those with smaller probabilities of being assigned to the dose plan they received are considered under-represented and are rewarded accordingly by assigning higher weights. Thus, the weights are inversely proportional to the probability of dose received. The estimated cumulative effect of dose A vs. B from such re-weighted data can be obtained by fitting a model that links potential outcome with a pre-specified dose regimen by simply adopting a variant of an "association model" that connects observed outcomes with observed dose history. Then estimated causal effect can be evaluated by comparing potential outcomes for the "hypothetical" population of patients who would be continuously exposed to dose A vs. B up to a given time point, even if contrary to the fact. The information gained from MSM analysis of flexible dose trials may not be adequate for conclusive demonstration of the dose relationship for a medication, but may be useful to assess the need for definitive fixed dose trials of a medication and to determine the best design of confirmatory fixed-dose studies.

Applying IPTW to flexible dose trials of antipsychotics suggested that there was no strong dose relationship for olanzapine. In contrast, unweighted analysis of dose relationships in these trials suggested negative dose responses, presumably because of the strong relationship between outcome and subsequent dose. Thus, IPTW appears to remove this bias from the estimated dose effect. The results of fixed dose studies of olanzapine are mixed in regards to dose relationship but not inconsistent with our findings. For olanzapine in the treatment of schizophrenia disorders, some studies have suggested a positive dose response [16,17], whereas other studies have not found a significant dose relationship [18,19]. There are no published fixed dose studies of olanzapine in patients with bipolar disorder that allow examination of dose relations.

This was an exploratory analysis with limited data. As any attempt to extract a causal relationship from observational data, MSM requires certain untestable assumptions: in addition to standard assumptions that the data analytic models (for selection bias and for the causal effect) were correctly specified, we have to assume that the model for selection bias accounts for all potential confounders (there are no unmeasured confounders). Additional sensitivity analyses (not reported here) suggested that the results remained stable under varying model specifications both for modeling probability of dose assignment and drop-out in construction of weights at the first step and for modeling causal effect on weighted data in Step 2. Briefly, for Step 1 only baseline severity (among pre-treatment covariates) and changes in outcome and to some extent occurrence of AEs (among post-treatment outcomes) affected dose assignments. This was consistent with our previous analysis of the same and other data sets [20]. Actually, AEs were a rather weak predictor that would have been eliminated had we used aggressive search algorithms with cross-validation or other model selection tools that target prediction error (as suggested in [21,22]). However, we left it in the model as we would rather overfit than underfit the model. In contrast to a less controlled observational study with many potential confounders [21], overall model selection for dose assignment was not the biggest issue in this study. Because we are considering longitudinal data with time-dependent confounders and the model naturally incorporated previous dose which is a strong predictor of subsequent dose, it acts as a natural model selection device, cutting off all weak predictors and leaving only strong predictors that can explain dose beyond previous dose. We also considered logistic regression models with time-varying intercept which proved unnecessary for our data, again probably because previous dose and other variables had absorbed any possible time trend in doses. Other variations on modeling the dose selection mechanism were using different dose intervals. For example, in the second study we first grouped doses in 3 intervals according to 10, 15, 20 mg and then into 2 intervals (10–15, 20). Although larger intervals would avoid the trouble of having zero probability associated with certain potential dose configuration, finer intervals may achieve better control over selection bias by modeling finer details of the dose selection process. This issue is related to the experimental treatment assignment (ETA) assumption that was emphasized and discussed by Mortimer *et al.*[21] and Petersen *et al.*[22]. In our analysis, the ETA assumption may have been partially violated in that some dose profiles (e.g. abruptly changing dose from 5 to 20 mg) would not be attainable in a flexible dose trial where protocols allow only one increment increase at a time. However, in the sensitivity analysis, the different dose groupings resulted in very similar analyses. In addition, we performed a separate simulation study using dose process as estimated from the data [13]. In this simulation study, we also had some probabilities close to 0 for abrupt changes of dose between two visit intervals. We obtained reasonably good results with almost unbiased estimates of overall causal effect.

Another difficulty in implementing IPTW methodology is that estimated weights (even when using their "stabilized" version) may be highly skewed by a few individuals with extreme values, requiring additional sensitivity analyses [4]. In our analyses of the bipolar trial, a few weights of very high magnitude (> 100) had unduly large impact on the estimates and we adopted models with such values removed or truncated (with very similar results). Further we tried to impose some truncation limit for stabilized weights requiring them not to exceed levels of 10, 20, and 50 also with very similar results. In the schizophrenia trial the weights were all within 0 to 5.

There are other challenges in evaluating causal effect from longitudinal data. The impact of IPTW and effectiveness of weight-adjusted analyses may vary across the duration of the clinical trial. Time-dependent IPTW may be more appropriate for bias reduction in the early phase when most dose titration due to efficacy/tolerability occurs, while more traditional analyses by stratified propensity score may be appropriate for later stages of treatment maintenance when dose adjustments are minimal. Evaluating potential dose effect may be difficult for the maintenance phase when most patients may be already receiving their "optimal" doses. Weighting requires that both under- and over-represented patients exist in the observed data; however, in clinical trials having a built-in deterministic dose escalation, some groups of patients may receive their actual dose with probability close to 1, while probabilities of certain pre-specified dose regimen of interest may be estimated as zero in these groups. In this situation, eliminating selection bias using IPTW may not be possible since the assumption of ETA is violated and other models are needed [3,23]. For a description of methods based on structural nested mean models see Brumback *et al.*[23]. Importantly, for the flexible dose trials investigated in the present paper, the protocols did not contain any deterministic or probabilistic rules except allowing dose reduction for AEs, at physician's discretion. This is consistent with the observation that intolerable AEs are rarely seen with atypical antipsychotics. Therefore, our strategy was to use IPTW MSM methodology to mimic potential outcomes that one would expect had patients been randomized to fixed dose groups. Although this estimation target may be considered as "gold standard" for evaluating efficacy of specific dose ranges for labeling purposes by the regulatory agencies, it may be somewhat questionable from the public health perspective. It may be more relevant from a public health perspective to evaluate potential outcomes associated with certain flexible dynamic regimens such as described in Murphy *et al.*[24]. Though one can argue that flexible dose trials, by their very nature, are implementing dynamic treatment regimes, in absence of clearly defined rules in the protocol, it appears that elicitation of such rules and estimating associated potential outcomes from flexible dose trials of relatively small size may pose difficulties. For a thorough discussion of estimating effects of dynamic treatment regimes see Murphy *et al.*[24].

Additionally, optimal dose for response measured by symptom reduction may not necessarily be the same as the optimal dose for relapse prevention and the applicability of this methodology to clinical trials involving other disorders such as depression or anxiety may be limited. Furthermore, the lack of a dose response relationship may be due to a variety of clinical factors that increase variation among individuals in drug response not routinely assessed in clinical trials such as differences in drug metabolism or neuro-receptor activities and possibly not adequately randomized in a relatively small group of study subjects. Finally, even though statistically significant dose-response effects were not found, this does not mean that significant effects are not present. It is possible that the unweighted approaches have more power than the IPTW approach, as the use of weights confers added variability to the analysis.

## Conclusion

IPTW may provide a methodology to estimate a probabilistic dose response relationship from flexible-dose clinical trials. This information could then be used in order to better design definitive fixed-dose studies. Simulations with a variety of datasets are needed to better assess the IPTW methodology for assessing dose response relationships.

## Competing interests

Authors IL, DHA, CM, DF, and JPH are employees and stockholders of Eli Lilly and Company.

## Authors' contributions

IL conceived the study and made substantial contributions to the analysis design, data analysis, and critical revision of the manuscript. DHA made substantial contributions to the interpretation of data, drafting, and critical revisions to the manuscript. CM, DF, DB, and JPH made critical revisions to the analysis plan and manuscript. All authors read and approved the final manuscript.

## Pre-publication history

The pre-publication history for this paper can be accessed here:


